# Lonely in Different Relationships: Bidirectional Effects between Parent- and Peer-Related Loneliness in Adolescence

**DOI:** 10.3390/ijerph19127014

**Published:** 2022-06-08

**Authors:** Flore Geukens, Annette Spithoven, Margot Bastin, Janne Vanhalst, Marlies Maes

**Affiliations:** 1 School Psychology and Development in Context, KU Leuven, 3000 Leuven, Belgium; awmspithoven@hotmail.com (A.S.); margot.bastin@kuleuven.be (M.B.); 2 Department of Developmental, Personality and Social Psychology, Ghent University, 9000 Ghent, Belgium; janne.vanhalst@ugent.be; 3 Youth Studies, Utrecht University, 3584 CH Utrecht, The Netherlands; m.maes@uu.nl

**Keywords:** peers, parents, loneliness, random intercept cross-lagged panel model

## Abstract

Although it is assumed that loneliness in one relationship might put one at risk of experiencing loneliness in another relationship, this association has rarely been examined as such. In this longitudinal study, we examined the associations between peer- and parent-related loneliness in a sample of 3391 adolescents across three waves (*M*_age Wave 1_ = 14.53; 59.3% female). Using random intercept cross-lagged panel models, parent- and peer-related loneliness were found to be stable over time and were concurrently related to each other. Moreover, the state of peer-related loneliness predicted the state of parent-related loneliness one year later. Thereby, the current study provides limited evidence of a carry-over effect between relation-specific types of loneliness.

## 1. Introduction

Adolescence is a developmental period in which individuals are particularly vulnerable to developing psychosocial problems, partly due to substantial changes in the social relationships of adolescents [1]. More specifically, in comparison with children, adolescents expect greater intimacy and self-disclosure from their social relationships; they engage in different and more diverse social situations and their main socializing partners change from parents to peers [2]. Hence, initiating and maintaining satisfying social relationships is seen as an important developmental task during adolescence [1]. Failing to fulfill this task may result in feelings of loneliness, which is a painful experience that has been found to be detrimentally related to the well-being of adolescents. For example, adolescents who feel lonely also experience more psychosocial problems, such as anxiety and depressive symptoms, and more physical problems, such as sleep problems and cardiovascular incidents [3,4,5]. As loneliness arises from unfulfilled relationship needs, people can experience loneliness in one relationship context, but not another [6]. Two important relationship contexts for adolescents are the peer context and the parent context. It has been suggested that relationship problems in one context may carry over to another context [7], but empirical evidence is largely lacking. If such carry-over effects exist, this would mean that loneliness feelings in one relationship could function as a barrier to build and maintain meaningful relationships with other people. In that case, this would also mean that clinicians should be aware that problems with relationships in one domain could lead to problems with relationships in other domains so that they can monitor and target this when needed. Although researchers increasingly acknowledge the importance of distinguishing loneliness types, the focus is still on general loneliness in the majority of studies. When associations between loneliness types are revealed over time, this would add to the literature, highlighting the importance of tackling loneliness in different relationships. In this study, we aimed to examine how parent-related loneliness is associated with peer-related loneliness over time in adolescence.

### 1.1. Parent- and Peer-Related Loneliness

Loneliness is defined as a negative emotional reaction to the experienced discrepancy between the perceived and desired quantity or quality of one’s social relationships [8]. Although several researchers have suggested that loneliness is a unidimensional phenomenon that varies in intensity [9], researchers have increasingly suggested that loneliness should be considered as a multidimensional phenomenon [10,11]. The multidimensional approach to loneliness is based on the premise that different relationships fulfill different social needs [11]. For example, parents are assumed to particularly provide nurturance and guidance whereas peers particularly provide a sense of integration [12]. When a certain type of relationship does not fulfill a particular social need, adolescents might experience loneliness within this relationship, but not in another relationship context.

One commonly made distinction of loneliness types is between emotional and social loneliness. Emotional loneliness is experienced when close emotional attachments are absent or lacking in quality. Social loneliness is experienced when a network of social relationships is absent or lacking in quality. Within each of these loneliness types, further subtypes can be identified depending on the relationship in which loneliness is experienced [13]. For instance, emotional loneliness can be experienced in relation to a parent or a best friend and social loneliness can be experienced in relation to the broader family or a group of friends. Previous research has indicated that relationship-specific experiences of loneliness are related, but are separate constructs [14]. Moreover, a recent study showed that parent- and peer-related loneliness develop in opposite directions during adolescence; that is, parent-related loneliness increases throughout adolescence whereas peer-related loneliness decreases [15]. In addition, research attempting to identify different subgroups of adolescents based on loneliness experiences in different relationships found separate groups, with one group of adolescents experiencing loneliness in the relationship with their parents without experiencing loneliness in relation to their peers and vice versa for another group [6]. These relation-specific loneliness experiences have also been found to be differentially related to various adjustment outcomes (e.g., [16]). For instance, peer-related loneliness was found to be related to social phobia whereas parent-related loneliness was related to self-harm [16]. These findings highlight the importance of distinguishing between loneliness experiences in various relationships.

### 1.2. Association between Parent- and Peer-Related Loneliness

Even though there is increasing evidence that different relation-specific types of loneliness can be distinguished, it is not fully understood how loneliness in one relationship relates to loneliness in other relationships. Based on the need–threat/need–fortification framework [17], two contrasting hypotheses can be proposed. On the one hand, it could be that higher levels of loneliness in one relationship type lead to lower levels of loneliness in another relationship type. When experiencing a thwarted need to belong (i.e., loneliness), individuals may act to fortify their relational needs, leading to prosocial thoughts and behaviors. In other words, when adolescents experience that one type of relationship is not fulfilling their relational needs, they might increase their efforts to establish another (type of) relationship that does fulfill their relational needs.

On the other hand, it can also be hypothesized that higher levels of loneliness in one relationship type lead to higher levels of loneliness in another relationship type. For example, it could be that experiencing ostracism, or experiencing being ignored or excluded, threatens the need of an individual for control and meaningful existence, leading to attempts to fortify their needs of control and recognition that might be dealt with through antisocial thoughts and behaviors [17]. Such thoughts and behaviors would then hinder the formation of fulfilling relationships with other people, leading to higher levels of loneliness in other relationship types as well.

This idea is in line with the socio-cognitive loneliness model [18], which suggests that loneliness experiences activate a series of cognitions to ensure self-preservation (e.g., hypervigilance for social threats). Paradoxically, these activated cognitions are biased in a way that social interactions are perceived as more negative and less positive, resulting in confirmatory behaviors that do not promote reconnection with others, but rather elicit negative reactions from others. These confirmatory behaviors in turn reinforce the initial cognitive bias. In this way, the loneliness levels are assumed to endure over time and could be generalized from one relationship to another [2,19]. For example, lonely individuals might withdraw from all social relationships as a result of previous negative interactions in one relationship, which might increase their feelings of loneliness as they become more socially isolated.

Empirical evidence testing these hypotheses is scarce. Based on cross-sectional work, a meta-analysis indicated that parent- and peer-related loneliness are positively correlated (*r* = 0.22; [19]). This positive correlation could suggest that experiencing parent-related loneliness puts one at risk of experiencing peer-related loneliness or, vice versa, that experiencing peer-related loneliness puts one at risk of experiencing parent-related loneliness. It is also possible that peer- and parent-related loneliness reciprocally influence one another, as suggested by transactional models of development [7], creating a vicious cycle. However, longitudinal studies including both peer- and parent-related loneliness are lacking. As a result, it remains unclear whether an association between different relation-specific types of loneliness can be found to hold over time and whether the associations are unidirectional or bidirectional.

### 1.3. The Current Study

In the current study, we adopted a transactional approach to loneliness as experienced in different types of social relationships. Specifically, we aimed to examine whether the cross-sectional association between parent- and peer-related loneliness found in previous studies held across time and within individuals. We examined the association between peer- and parent-related loneliness in regard to state-like fluctuations over time (i.e., within-person effects) above and beyond trait-like tendencies (i.e., between-person effects; [20]); that is, within-person processes were separated from stable between-person differences [20]. We hypothesized that experiencing parent-related loneliness at Time *N* would be related to experiencing more peer-related loneliness at Time *N* + 1 within individuals, and vice versa.

## 2. Method

### 2.1. Procedure and Participants 

In this study, we combined data from three independent samples from the Dutch-speaking part of Belgium [21,22,23,24]. In each of the three studies, the same procedure was followed; that is, passive consent was obtained from parents and active consent was obtained from adolescents. The design of the three longitudinal studies was also comparable with a one-year interval between assessments. In each study, during three subsequent years, adolescents completed questionnaires during regular school hours. 

The first sample, which was part of another larger longitudinal dataset [23], consisted of 551 adolescents (62.80% female) aged between 12 and 17 years old at the first assessment (*M* = 14.82, *SD* = 0.79). In this sample, 21.80% of the adolescents did not participate in Wave 2 and 24.90% did not participate in Wave 3. The MCAR Test of Little [25] revealed a normed χ^2^ of 1.16, which indicated that the data were randomly missing (i.e., the normed χ^2^ was below 3; [26]).

The second sample consisted of 1371 adolescents (53.20% female) aged between 11 and 17 years old at the first assessment (*M* = 13.21, *SD* = 0.79) who participated in a larger longitudinal study [21,22]. Of these 1371 adolescents, 25.50% did not participate in Wave 2, and 38.80% did not participate in Wave 3. The MCAR Test if Little [25], using an expectation maximization estimation, revealed a normed χ^2^ of 1.30, which indicated that the data were randomly missing (i.e., the normed χ^2^ was below 3; [26]).

For the third sample, data from 1469 adolescents (63.80% female) aged between 11 and 20 years old (*M* = 15.64, *SD* = 1.33) were used from a larger longitudinal study [24]. Of this sample, 65.40% were enrolled from the first assessment onwards, of which 17.77% did not participate in the second assessment and 38.97% did not participate in the third assessment. In addition, 23.23% enrolled at the second assessment, of which 39.00% did not participate in the third assessment. The remaining adolescents (11.37%) of the total sample enrolled at the third assessment. The MCAR Test of Little [25] revealed a normed χ^2^ of 1.08, which indicated that the data were randomly missing (i.e., the normed χ^2^ was below 3; [26]).

Combining these three samples resulted in a sample of 3391 adolescents (59.4% female) aged between 11 and 20 years old at the first assessment (*M* = 14.52, *SD* = 1.67). The MCAR test of Little [25] on this combined sample, using an expectation maximization, revealed a normed χ^2^ of 1.23, which indicated that the data were randomly missing (i.e., the normed χ^2^ was below 3; [26]). Given the variation in age at the first assessment both within and across the studies, age was included as a covariate in our analyses.

### 2.2. Measures

Peer- and parent-related loneliness were measured with the Loneliness and Aloneness Scale for Children and Adolescents (LACA; [10]). Both subscales consist of 12 items. Example items from the peer-related loneliness subscale are “I think I have fewer friends than others” and “I feel excluded by my classmates”. Example items from the parent-related loneliness subscale are “I feel left out by my parents” and “I find it hard to talk to my parents”. The items were rated on a four-point Likert scale, ranging from 1 (never) to 4 (often). For each participant, an individual mean score was calculated, resulting in mean scores ranging from 1 (low loneliness) to 4 (high loneliness). The questionnaire has been shown to be a valid [10] and reliable measure of loneliness in various countries [27].

### 2.3. Statistical Analysis

A random intercept cross-lagged panel model (RI-CLPM) was estimated with M*plus* Version 8.2 [28]. This type of model allowed us to examine the longitudinal associations between peer- and parent-related loneliness whilst taking into account the stability of the constructs and their within-time correlations. Given that the data were randomly missing, a full information maximum likelihood estimation (FIML) was used to account for the missing data. A robust maximum likelihood estimation (MLR) was used to account for the non-normality of the data.

In an RI-CLPM, the observed scores are decomposed into two parts. The within-person processes are separated from the stable between-person differences [20]. First, all observed scores of peer-related loneliness are regressed on a single latent factor and all observed scores of parent-related loneliness are regressed on another single latent factor [20]; that is, a random intercept for peer-related loneliness and a random intercept for parent-related loneliness are specified. These two random intercepts represent reliable time invariant differences between individuals and can, therefore, be interpreted as trait-like factors. Second, each observed loneliness score (i.e., one peer-related loneliness score and one parent-related loneliness score for each individual from each assessment wave) is regressed on its own latent factor [20]. Across the three waves, this results in three peer-related loneliness latent factors and three parent-related loneliness latent factors. These six latent factors represent situation-dependent fluctuations in loneliness within an individual and can, therefore, be interpreted as state-like factors.

Correlations, stability, and cross-lagged paths can then be specified between these state-like latent factors. First, we estimated an unconstrained model in which all of these aforementioned paths were freely estimated (i.e., Model 1). Second, more parsimonious models were estimated and compared with the first unconstrained model; that is, the stability coefficients of peer-related loneliness (i.e., from peer-related loneliness at Wave *N* to peer-related loneliness at Wave *N* + 1) were constrained to be equal to one another in Model 2. In Model 3, the stability coefficients of parent-related loneliness (i.e., from parent-related loneliness at Wave *N* to parent-related loneliness at Wave *N* + 1) were constrained to be equal. In Model 4, the within-time correlations between the state-like factors of both peer- and parent-related loneliness at Wave 2 and Wave 3 were constrained to be equal to one another. In Model 5, the cross-lagged paths from parent-related loneliness to peer-related loneliness were constrained to be equal to one another. In Model 6, the cross-lagged paths from peer-related loneliness to parent-related loneliness were constrained to be equal. The path coefficients of the best fitting model were interpreted. In all models ‘age’ was added as a predictor to the observed scores for both peer- and parent-related loneliness at each measurement occasion in order to control for the variability in age at the different measurement waves. This path was set to be equal for each measurement occasion in all models as we did not expect this effect to change across the measurement waves [29]. The syntax of the different models are provided on the Open Science Framework (https://osf.io/k43r2/?view_only=d00105049a964c64a6090c98ff693541 accessed on 17 May 2022).

The model fit was considered to be satisfactory if the Satorra–Bentler (SB)-scaled chi^2^ was non-significant [30]. In addition, the root mean square error of approximation (RMSEA) was designated as 0.06 or smaller, the standardized root mean square residual (SRMR) was designated as 0.08 or smaller, and the comparative fit index (CFI) exceeded 0.95 [31]. When comparing the model fit of the different models, the model with the lowest RMSEA and SRMR as well as the highest CFI was preferred [32]. Additionally, a significant chi-squared difference test indicated that the models differed from one another; the more complex model (i.e., the model with the least constraints) was preferred [30]. The paths in the preferred model were investigated and path coefficients with *p*-values below 0.05 were considered to be statistically significant.

## 3. Results

### 3.1. Descriptive Statistics

Table 1 summarizes the correlations, means, standard deviations, and Cronbach’s alphas for peer- and parent-related loneliness across the three measurement waves. Cronbach’s alphas showed a good reliability for both subscales at each measurement occasion. High positive correlations were found for parent-related loneliness on three measurement occasions, suggesting the relative stability of parent-related loneliness across time. Similarly, high positive correlations were found for peer-related loneliness on various measurement occasions. There were weak to moderate positive correlations between parent- and peer-related loneliness on different measurement occasions. The correlations between parent- and peer-related loneliness were in line with the reported estimated mean correlation in a meta-analysis on this regard (i.e., *r* = 0.22; [27]).

### 3.2. Random Intercept Cross-Lagged Panel Models

The model fit of the different RI-CLPMs as well as the model comparisons are shown in Table 2. All models showed a good fit. The model with the stability paths for peer-related loneliness constrained to be equal (i.e., Model 2) was not preferred over the fully unconstrained model (i.e., Model 1). Model 3, with the stability paths of parent-related loneliness constrained to be equal, was not preferred over Model 1. The model in which the within-time correlations between parent- and peer-related loneliness at Waves 2 and 3 (i.e., Model 4) were constrained to be equal was preferred over Model 1. The model in which the paths from parent-related loneliness to peer-related loneliness one year later were constrained to be equal was also preferred over Model 1. The model in which the paths from peer-related loneliness to parent-related loneliness one year later were constrained to be equal was not preferred over Model 1. As the constraints of Models 4 and 5 were allowed, we compared a final model in which we applied the constraints of Models 4 and 5 simultaneously (i.e., Model 7) to the fully unconstrained model (i.e., Model 1). This comparison showed that Model 7 was preferred over Model 1.

**Figure 1:**
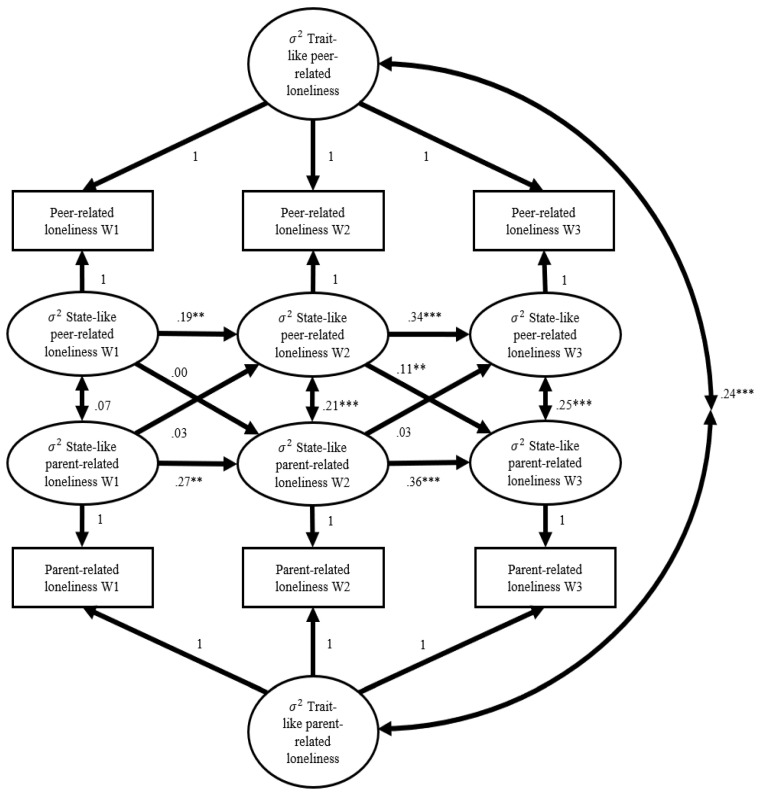
Random intercept cross-lagged model with standardized path coefficients from the meta-analysis. W: Wave. ** *p* = 0.01, *** *p* < 0.001.

The standardized path coefficients of Model 7 are shown in Figure 1 (for the unstandardized coefficients, standard errors, and *p*-values, see Table 3). First, the trait-like factors (i.e., the random intercepts) of parent-related loneliness and peer-related loneliness were moderately correlated. This indicated that the adolescents who reported more parent-related loneliness than their peers across the three measurement occasions also reported more peer-related loneliness than their peers across these measurement occasions and vice versa. Second, the moderate stability coefficients indicated that the earlier state of parent- and peer-related loneliness predicted, respectively, the state of parent- and peer-related loneliness one year later. Third, the state of peer-related loneliness at Wave 2 was related to the state of parent-related loneliness at Wave 3. Specifically, the adolescents who reported higher levels of peer-related loneliness at Wave 2 were more likely to report higher levels of parent-related loneliness at Wave 3. Remarkably, this effect was not found between the state of peer-related loneliness at Wave 1 and the state of parent-related loneliness at Wave 2. Fourth, the state of parent-related loneliness was not related to the state of peer-related loneliness one year later. Fifth, cross-sectionally, the state of peer-related loneliness was related more to the state of parent-related loneliness and vice versa. Finally, age was a significant positive predictor for the state of parent-related loneliness at all waves; that is, higher ages were more associated with the state of parent-related loneliness.

## 4. Discussion

Researchers adhering to a multidimensional approach on loneliness underscore the importance of distinguishing between loneliness experiences in various relationships (e.g., [10,11,16]). The moderate positive correlation between peer- and parent-related loneliness found in a previous work [27] suggests that experiencing loneliness in one relationship might put one at risk of also experiencing loneliness in other relationships. On the other hand, based on the need–threat/need–fortification model [17], a negative association between loneliness in the two relationship types could exist as well. However, studies longitudinally examining this hypothesis are lacking, and it remains unclear whether the associations among different relation-specific types of loneliness are unidirectional or bidirectional. The current study examined how loneliness experienced in the relationship with parents on the one hand and peers on the other hand is related to each over time.

The findings indicated that state-like loneliness, both in relation to parents and in relation to peers, endures over time. More specifically, the stability of peer- and parent-related loneliness in adolescents over time was not fully accounted for by their respective trait-like loneliness components. Similar results were found for general loneliness experiences of adults in a previous study of Lim et al. (2016; [33]) in which the stability of general loneliness was not fully accounted for by the trait-like loneliness component either. These findings suggest that loneliness is susceptible to changes due to momentary external factors and that momentary fluctuations in loneliness might affect future levels of loneliness.

The current study found limited evidence of a carry-over effect between parent- and peer-related loneliness. First, we found an effect of peer-related loneliness on parent-related loneliness one year later; that is, adolescents who experienced more peer-related loneliness were more likely to experience increased parent-related loneliness one year later. However, this cross-lagged effect was not found at all time points and it was very small. Hence, we are hesitant to interpret this effect. Second, a correlated change between parent- and peer-related loneliness was found; that is, changes in the state of parent-related loneliness were associated with changes in peer-related loneliness in the same direction. Our findings seem to suggest that the reciprocal process in which relation-specific loneliness types influence each other does not operate over years, but might occur across a smaller time interval. We encourage future studies to examine this hypothesis.

### Strengths and Limitations

The current study had several strengths such as the use of a large longitudinal dataset, the use of a well-established loneliness measure, and the application of state-of-the art statistical techniques. However, this study was not without limitations. First, although the cross-lagged panel models included a temporal dimension, no causal conclusions could be drawn as this was still a correlational method.

Second, we only relied on self-reports to measure parent- and peer-related loneliness. Although this method has been put forward as the most adequate assessment method for a subjective and internal experience such as loneliness [3,34], this approach might have resulted in inflated findings due to common-method variances.

Third, although we examined loneliness in relation to key figures in the social environment of adolescents, examining loneliness in relation to additional significant others in the lives of adolescents is important as well. For instance, romantic relationships become increasingly important in adolescence and are associated with the well-being of adolescents [35]. Hence, a broader examination of the carry-over effects of loneliness across different relationship types in adolescence is necessary.

Fourth, in this study we used a community sample in which loneliness levels were naturally not that high. In line with other research using community samples, the distribution of loneliness was skewed, with the majority of participants scoring below 2 [10]. We urge readers to take this issue into account when interpreting our results. Future research could replicate this study in a clinical sample. Nonetheless, we do not expect the associations between the variables to be substantially different among adolescents with high loneliness scores.

Finally, the sample of the current study varied in terms of age; that is, at the first measurement occasion, adolescents between the ages of 11 and 17 years old participated in this study. Although we corrected for age with regard to the mean levels of both types of loneliness, we could not verify whether the correlations and regression coefficients were different across adolescence. Future research should examine this research question whilst taking the different stages of adolescence into account.

## 5. Conclusions

The findings of the current study indicated that, in line with previous research, parent- and peer-related loneliness were positively associated with one another at the same point in time. Moreover, a significant longitudinal within-person effect was found, indicating that the state of peer-related loneliness predicted the state of parent-related loneliness one year later. Hence, the current study provides limited evidence of a carry-over effect from peer-related loneliness to parent-related loneliness. Although further research is needed, our findings suggest that high levels of a relation-specific loneliness type could be a risk factor for another relation-specific loneliness type.

## Figures and Tables

**Table 1:** Correlations among study variables.

Variable	α	*M*	*SD*	1	2	3	4	5
1. Parent W1	0.90	1.70	0.58	-				
2. Parent W2	0.92	1.67	0.59	0.68 ***	-			
3. Parent W3	0.92	1.64	0.57	0.61 ***	0.72 ***	-		
4. Peer W1	0.89	1.64	0.55	0.21 ***	0.19 **	0.18 *	-	
5. Peer W2	0.90	1.63	0.55	0.19 ***	0.26 ***	0.25 **	0.56 ***	-
6. Peer W3	0.90	1.62	0.54	0.18 ***	0.22 ***	0.31 ***	0.49 ***	0.63 ***

*N* = 3391. W1: Wave 1. W2: Wave 2. W3: Wave 3. * *p* < 0.05. ** *p* < 0.01. *** *p* < 0.001.

**Table 2:** Model fit of the random intercept cross-lagged panel models of peer- and parent-related loneliness.

Model	RMSEA	SRMR	CFI	χ^2^	*df*	Δχ^2^	*df*
1	0.023	0.010	0.998	13.842 *	5		
2	0.030	0.020	0.995	24.066 ***	6	10.121 **	1
3	0.025	0.015	0.996	18.758 **	6	4.834 *	1
4.2	0.020	0.010	0.998	14.021 *	6	0.209	1
5.2	0.019	0.010	0.998	13.713 *	6	0.258	1
6.2	0.025	0.013	0.997	18.278	6	4.418 *	1
7	0.017	0.010	0.998	13.640 *	7	0.326	2

χ^2^: Satorra–Bentler χ^2^. *N* = 3391. Chi^2^ difference test was always performed in comparison with the preferred model in the step before. * *p* < 0.05; ** *p* < 0.01; *** *p* < 0.001.

**Table 3:** Path coefficients of the random intercept cross-lagged panel Model 7 of parent- and peer-related loneliness.

Path	*B*	*SE*	*β*	*p*
Parent W1 à Parent W2	0.27	0.08	0.24	0.002
Parent W2 à Parent W3	0.36	0.06	0.36	<0.001
Peer W1 à Peer W2	0.19	0.06	0.19	0.001
Peer W2 à Peer W3	0.33	0.05	0.34	<0.001
Parent W1 à Peer W2	0.03	0.04	0.03	0.471
Parent W2 à Peer W3	0.03	0.04	0.03	0.471
Peer W1 à Parent W2	0.00	0.04	0.00	0.983
Peer W2 à Parent W3	0.11	0.04	0.11	0.005
Age à Parent W1	0.05	0.01	0.15	<0.001
Age à Parent W2	0.05	0.01	0.15	<0.001
Age à Parent W3	0.05	0.01	0.15	<0.001
Age à Peer W1	0.00	0.01	0.00	0.891
Age à Peer W2	0.00	0.01	0.00	0.891
Age à Peer W3	0.00	0.01	0.00	0.891
Parent W1—Peer W1	0.01	0.01	0.07	0.165
Parent W2—Peer W2	0.03	0.00	0.21	<0.001
Parent W3—Peer W3	0.03	0.00	0.25	<0.001
RI Parent—RI Peer	0.06	0.01	0.24	<0.001

*N* = 3391. Parent: parent-related loneliness. Peer: peer-related loneliness. W1: Wave 1. W2: Wave 2. W3: Wave 3. RI: random intercept. SE: standard error. à represents regression paths.—represents correlations.
